# Supplementary material for: Comprehensive Annotation and Functional Exploration of MicroRNAs in Lettuce
Source: Front Plant Sci. 2021 Dec 24;12:781836. doi: 10.3389/fpls.2021.781836 (PMC8739914; doi:10.3389/fpls.2021.781836)
Supplement: Supplementary Data 1 — Results of categories 0–2 from PARE-Seq analysis (including three subfiles:1_1, 1_2, 1_3). [file Data_Sheet_10.ZIP › GSM2230755.plot/Lsa-miRN69_Lsat_1_v5_gn_8_150760.1_1975_TPlot.pdf]

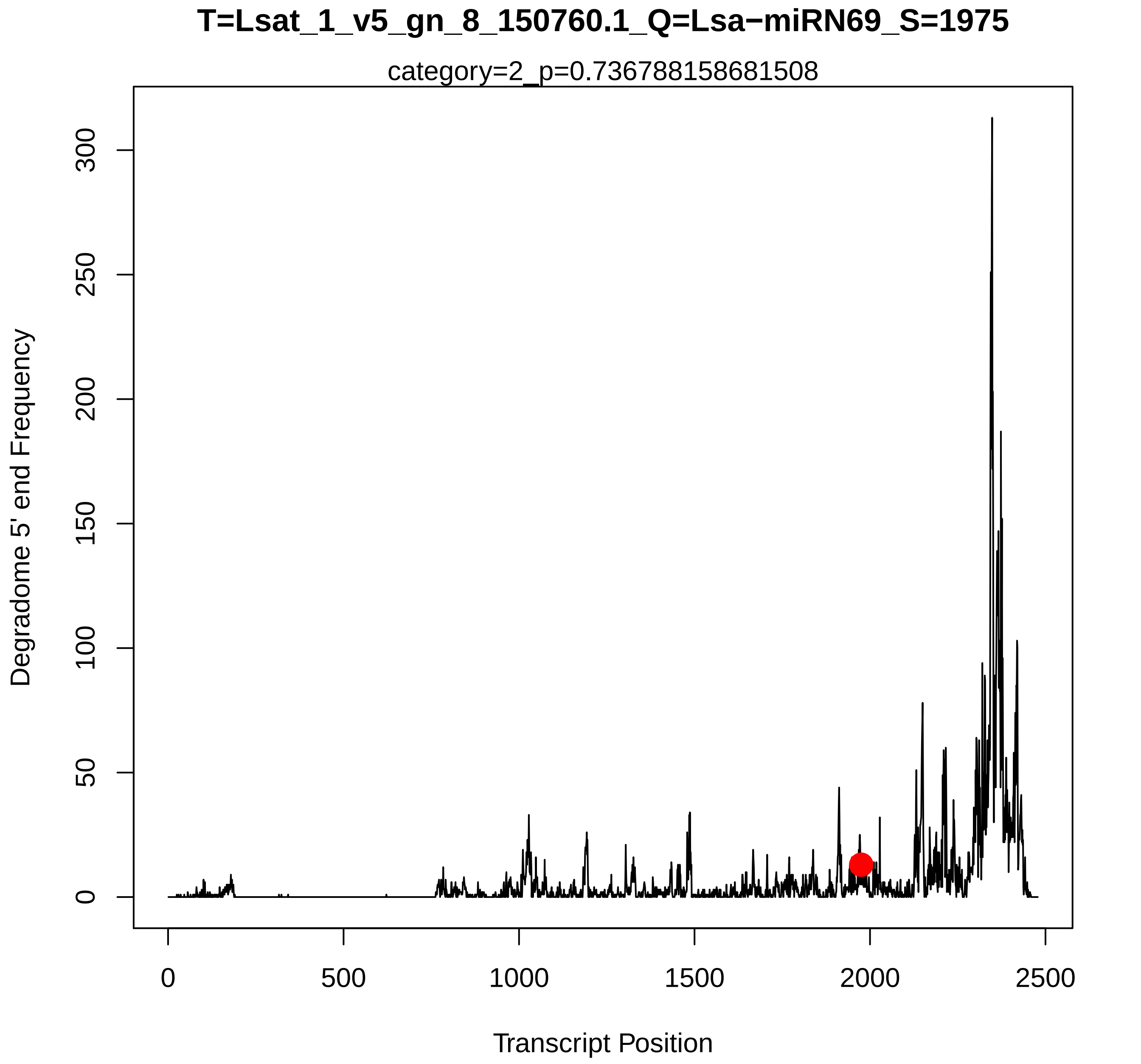
T=Lsat_1_v5_gn_8_150760.1_Q=Lsa−miRN69_S=1975
category=2_p=0.736788158681508
Degradome 5' end Frequency
0
50
100
150
200
250
300
0
500
1000
1500
2000
2500
Transcript Position
